# The stone-degrading metabolic potential of the urinary microbiome: Sexual dimorphism and dysbiosis in urolithiasis

**DOI:** 10.3934/microbiol.2026026

**Published:** 2026-09-03

**Authors:** Ying Li, Chengqiang Mo, Yun Xie, Zhixin Huang, Lingwu Chen, Xinqiang Xie, Junhang Luo, Shixuan Huang, Shuangjian Jiang, Tianhao Zhang, Jumei Zhang, Rongpei Wu, Qingping Wu

**Affiliations:** 1 State Key Laboratory of Applied Microbiology Southern China, Guangdong Provincial Key Laboratory of Microbial Safety and Health, National Health Commission Science and Technology Innovation Platform for Nutrition and Safety of Microbial Food, Key Laboratory of Big Data Technologies for Food Microbiological Safety, State Administration for Market Regulation, Institute of Microbiology, Guangdong Academy of Sciences, Guangzhou, 510070, China; 2 Department of Urology, The First Affiliated Hospital of Sun Yat-Sen University, Guangzhou, 510080, China; 3 Division of Gastrointestinal Surgery Center, The First Affiliated Hospital of Sun Yat-Sen University, Guangzhou, 510080, China; 4 Food and Drug Laboratory, Guangdong Detection Center of Microbiology, Guangzhou, 510070, China

**Keywords:** urinary microbiome, sex discrepancy, *Lactobacillus*, urolithiasis, urolith degradation

## Abstract

Urolithiasis is a prevalent global health burden characterized by high recurrence rates and marked sexual dimorphism. While the gut microbiome's role in oxalate degradation is established, the functional capacity of the local urinary microbiome remains largely unexplored. This study investigated the composition and function of the urinary microbiome in 40 healthy volunteers and 61 urolithiasis patients using 16S rRNA amplicon sequencing, alongside a retrospective analysis of stone composition in 2027 patients. We identified a diverse, ecologically structured microbiome in healthy urine dominated by genera such as *Lactobacillus*, *Streptococcus*, and *Gardnerella*. Significant sex-dependent differences were observed: While males exhibited higher microbial α-diversity, females demonstrated a distinct compositional structure with superior predicted functional capacity for stone degradation. Specifically, the female microbiome was enriched with enzymes including oxalyl-CoA decarboxylase, formyl-CoA transferase, urate oxidase, and purine nucleosidase. Correlation analysis identified *Lactobacillus*, *Enterococcus*, and *Gardnerella* as key contributors to this litholytic potential. Conversely, urolithiasis patients displayed significant dysbiosis, characterized by disrupted microbial networks and a marked reduction in these stone-degrading enzymes. Clinical data supported these findings, revealing a significantly higher prevalence of uric acid stones in males, consistent with their lower microbial degradation capacity. These findings suggest that the healthy urinary microbiome functions as a metabolically active defense system against stone formation. The observed sex-specific variations in microbial composition and enzymatic function provide a novel mechanistic explanation for the epidemiological sex disparities in urolithiasis, highlighting the urinary–kidney axis as a potential therapeutic target.

## 1. Introduction

Urolithiasis is a global health burden affecting approximately 10% of the population, with a recurrence rate that poses significant challenges to clinical management [1],[2]. The formation of urinary calculi is traditionally attributed to the supersaturation of stone-forming substances, primarily calcium oxalate and uric acid, derived largely from dietary sources and metabolic processes [3]. While human cells lack the enzymatic machinery to degrade these crystalline precursors, the human microbiome has emerged as a critical regulator of mineral homeostasis [4].

In the gastrointestinal tract, specific bacteria such as *Oxalobacter formigenes* are known to degrade oxalate, thereby reducing intestinal absorption and urinary excretion [5],[6]. However, the role of the local urinary microbiome remains largely unexplored. Historically, the healthy urinary tract was considered sterile; however, recent culture-independent sequencing advances have confirmed the presence of a resident urinary microbiota [7]. Despite this recognition, it remains unclear whether these local microbial communities, forming a urinary–kidney axis, possess the functional enzymatic capacity to actively participate in mineral homeostasis and degrade lithogenic substances, or how dysbiosis within this local environment contributes to stone formation.

Furthermore, urolithiasis exhibits marked sexual dimorphism, with a significantly higher incidence in men than in women [8]. While hormonal and lifestyle factors are often cited [9], the potential contribution of sex-specific microbial ecology has been overlooked. If the urinary microbiome acts as a protective barrier against stone formation, sex-based differences in microbial composition and enzymatic function could offer a novel explanation for this epidemiological disparity.

In this study, we employed an optimized *16S rRNA* amplicon sequencing protocol to overcome low-biomass limitations and characterize the urinary microbiome in healthy volunteers and urolithiasis patients. By mapping the ecological architecture of the healthy urinary microbiome, we sought to uncover its metabolic potential for degrading calcium oxalate and uric acid. Concurrently, we examined the relationship between sex-specific microbial signatures and clinical stone composition to provide a mechanistic understanding of the sex disparities observed in stone formation. We hope to provide new insights into the urinary–kidney axis and suggest that the urinary microbiome is a metabolically active defense system against urolithiasis.

## 2. Materials and methods

### 2.1. Study participants and sample collection

A total of 40 healthy volunteers (20 males, 20 females) and 61 patients with upper urinary tract calculi (32 males, 29 females) were recruited for urine microbiome analysis from the Department of Urology, The First Affiliated Hospital of Sun Yat-Sen University, between March and September 2021. Additionally, a retrospective analysis of stone composition was conducted on 2027 urolithiasis patients treated surgically at the same center from March 2018 to March 2021.

For the microbiome analysis, inclusion criteria were: (i) age > 18 years; (ii) diagnosis of upper urinary tract calculi confirmed by ultrasound within 2 weeks; (iii) no congenital urinary tract deformities; (iv) no history of malignancy, autoimmune disease, or diabetes; (v) for females, not pregnant and not menstruating during sampling; (vi) no symptoms or clinical evidence of urinary infection; and (vii) no antibiotic exposure (systemic or topical) within the past 2 months.

For the stone component analysis, inclusion criteria were: (i) age > 18 years; (ii) diagnosis of upper tract urolithiasis by ultrasound or CT; and (iii) receipt of surgical treatment following European Association of Urology (EAU) guidelines [10].

This study was approved by the Ethics Committee of the First Affiliated Hospital of Sun Yat-sen University (project [2021]481). Written informed consent was obtained from all participants.

### 2.2. Urinary stone component analysis

Stone components were analyzed using the LIIR-20 Automatic Infrared Spectrum Analysis System (Tianjin, China) and chemical analysis following Siener [11]. Stones were categorized according to EAU guidelines into calcium oxalate, uric acid, carbonate apatite, ammonium magnesium phosphate, cystine, and ammonium urate [12].

### 2.3. Urinary microbial DNA extraction and 16S rRNA amplification

Thirty milliliters of first-catch urine samples collected over three consecutive days were pooled and centrifuged at 16,000 × g for 10 minutes at 4 °C. Pellets were washed twice with 2 mL of PBS. This increased sample volume, compared to standard small-aliquot studies, effectively overcame the limitations of low urinary microbial biomass, enabling successful extraction of bacterial DNA and downstream microbiome analysis in all study participants. Microbial DNA was extracted using the QIAamp PowerFecal Pro DNA kit (Qiagen, Hilden, Germany). DNA concentration and purity were assessed using a Nanodrop spectrophotometer (Thermo Scientific, Waltham, MA, USA) and 0.6% agarose gel electrophoresis. High-integrity DNA was diluted to 20 ng/μL for amplification.

The V3–V4 hypervariable region of the *16S rRNA* gene was amplified using primers 341F (5′-CCTACGGGNGGCWGCAG-3′) and 806R (5′-GGACTACHVGGGTWTCTAAT-3′) (18) with the UCP Multiplex PCR Kit (Qiagen). Cycling conditions were: 95 °C for 3 min; 30 cycles of 95 °C for 30 s, 55 °C for 30 s, and 72 °C for 30 s; and a final extension at 72 °C for 5 min. PCR products were purified using AMPure XP beads (Beckman, Los Angeles, CA, USA) prior to library generation with the QIAseq Ultralow Input Library Kit (Qiagen).

### 2.4. Sequencing and data processing

Libraries were validated using an Agilent 2100 Bioanalyzer (Agilent Technologies, Santa Clara, CA, USA) and Qubit™ dsDNA HS Assay Kit (Thermo Scientific). Paired-end sequencing (2 × 300 bp) was performed on the Illumina MiSeq platform using the MiSeq Reagent Kit v3.

Raw data were demultiplexed using MiSeq Reporter. Primary FASTQ files were trimmed and quality-filtered using the Microbial Genomics Module in CLC Genomic Workbench 20.0 (Qiagen). Sequences were mapped to the Greengenes 13.5 database with a 97% similarity threshold for Operational Taxonomic Unit (OTU) generation.

### 2.5. Bioinformatics and statistical analysis

Taxonomic and statistical analyses were performed using MicrobiomeAnalyst [13]. The core microbiome was identified following Custer [14]. Network analysis was conducted in Calypso to identify co-occurring and mutually exclusive taxa using Pearson's correlation. Networks were visualized using Cytoscape v3.7.2 [15], with nodes representing genera (sized by relative abundance) and edges representing significant correlations (*P* < 0.05).

Metagenome functional prediction was performed using PICRUSt [16]. Pearson's correlation tested associations between key genera and urolithiasis-related metabolites. Differences in metabolites and KEGG pathways were analyzed using the Wilcoxon Rank test and visualized with GraphPad Prism v7 (GraphPad Software, San Diego, CA, USA).

Alpha diversity was assessed via ANOVA. Multivariate analysis of community structure was performed using redundancy analysis (RDA). Sex-specific taxonomic biomarkers were identified using linear discriminant analysis effect size (LEfSe) [17]. Statistical significance was defined as *P* < 0.05, with P-values adjusted for multiple comparisons using false discovery rate (FDR), Bonferroni, or AUC correction where appropriate.

## 3. Results

### 3.1. An ecologically structured urinary microbiome defines the baseline of urological health

Contrary to the traditional view of urinary sterility, diverse microbial communities were consistently detected across healthy individuals. At the phylum level, the urinary microbiome consisted of *Firmicutes*, *Actinobacteria*, *Proteobacteria*, *Bacteroidetes*, *Fusobacteria*, *Thermi*, *Tenericutes*, *TM7*, *Cyanobacteria*, *Acidobacteria*, and *Synergistetes* (Figure 1A). At the genus level, a structured community composed of dominant and low-abundance taxa was observed (Figure 1B), including *Lactobacillus*, *Streptococcus*, *Corynebacterium*, *Pseudomonas*, *Gardnerella*, *Enterococcus*, *Finegoldia*, *Peptoniphilus*, *Veillonella*, and *Anaerococcus*, indicating ecological organization rather than stochastic contamination.

The co-occurring analysis of urinary microbiome revealed an intricate network among the key microorganisms in the urinary tract: *Lactobacillus* exhibited antagonistic relations with *Streptococcus*, *Corynebacterium*, and *Finegoldia*, while mutual promotive relations were observed in *Streptococcus*, *Enterococcus*, *Corynebacterium*, and *Acinetobacter*, indicating potential ecological “hubs” that may contribute to microbiome stability (Figure 1C).

**Figure 1.**
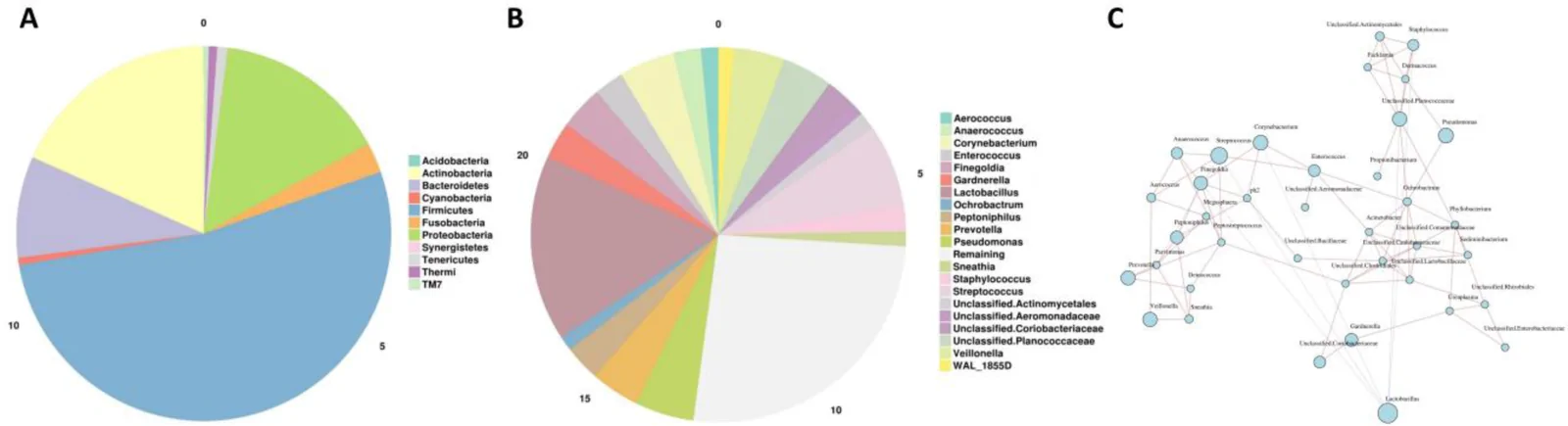
Composition and concurrent network of the healthy urinary microbiome. A: Composition of the healthy urinary microbiome at phylum level. B: Composition of the healthy urinary microbiome at the genus level. C: Concurrent network of the healthy urinary microbiome.

### 3.2. Sex-dependent divergence in microbial diversity and composition defines distinct urobiome states

Further investigation was carried out to confirm whether host sex influences urinary microbiome composition. Alpha diversity analysis demonstrated significantly higher genus-level diversity in males compared with females (*P* = 0.013; Figure 2A), indicating increased microbial richness and evenness in the male urinary tract. Furthermore, RDA revealed clear compositional segregation between male and female microbiomes (*P* < 0.001; Figure 2B), suggesting that sex is a major determinant of urinary microbial structure.

The dot-plot visualization of dominant genera further confirmed differential relative abundances between sexes (Figure 2C). LEfSe analysis identified sex-enriched genera (Figure 2D), with female-specific taxa and male-specific taxa forming distinct compositional signatures.

Importantly, functional prediction using PICRUSt revealed sex-associated metabolic divergence. At the KEGG second hierarchy level, multiple functional pathways differed significantly (Figure 3A). Notably, the female urinary microorganisms possessed a stronger ability in calcium oxalate degradation, with a higher RA of oxalyl-CoA decarboxylase (0.33‰ ± 0.39‰ in females vs 0.11‰ ± 0.28‰ in males, *P* = 0.011) and similar RA of formyl-CoA transferase (0.39‰ ± 0.42‰ in females vs 0.26‰ ± 0.28‰ in males, *P* = 0.449) (Figure 3B,C). Furthermore, better degradation of uric acid was also observed in the female urinary microorganisms, with both higher RAs of urate oxidase (0.0034‰ ± 0.0061‰ in females vs 0.0062‰ ± 0.0107‰ in males, *P* = 0.028) and purine nucleosidase (0.64‰ ± 0.40‰ in females vs 0.45‰ ± 0.58‰ in males, *P* = 0.029) (Figure 3D,E).

**Figure 2.**
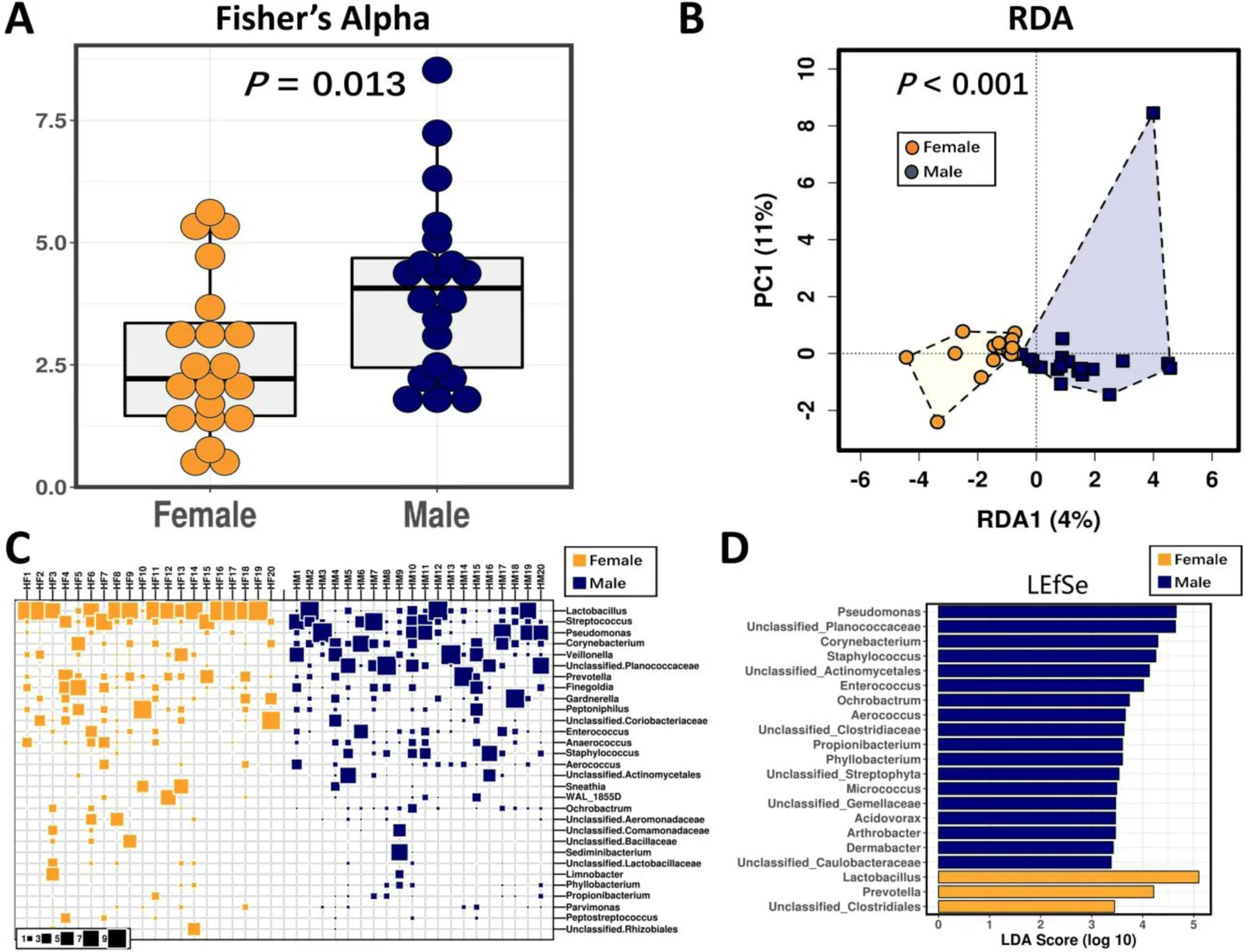
Sex-dependent differences in urinary microbiome composition and function. A: The male urinary microbiome exhibited significantly higher α-diversity at the genus level compared with the female microbiome. B: RDA demonstrated distinct compositional structures between male and female urinary microbiomes. C: LEfSe identified sex-specific genera within the urinary microbiome. D: Dot plot of the 30 dominant genera of both females and males in the urinary microbiome; dot size represents the relative abundance of each genus.

**Figure 3.**
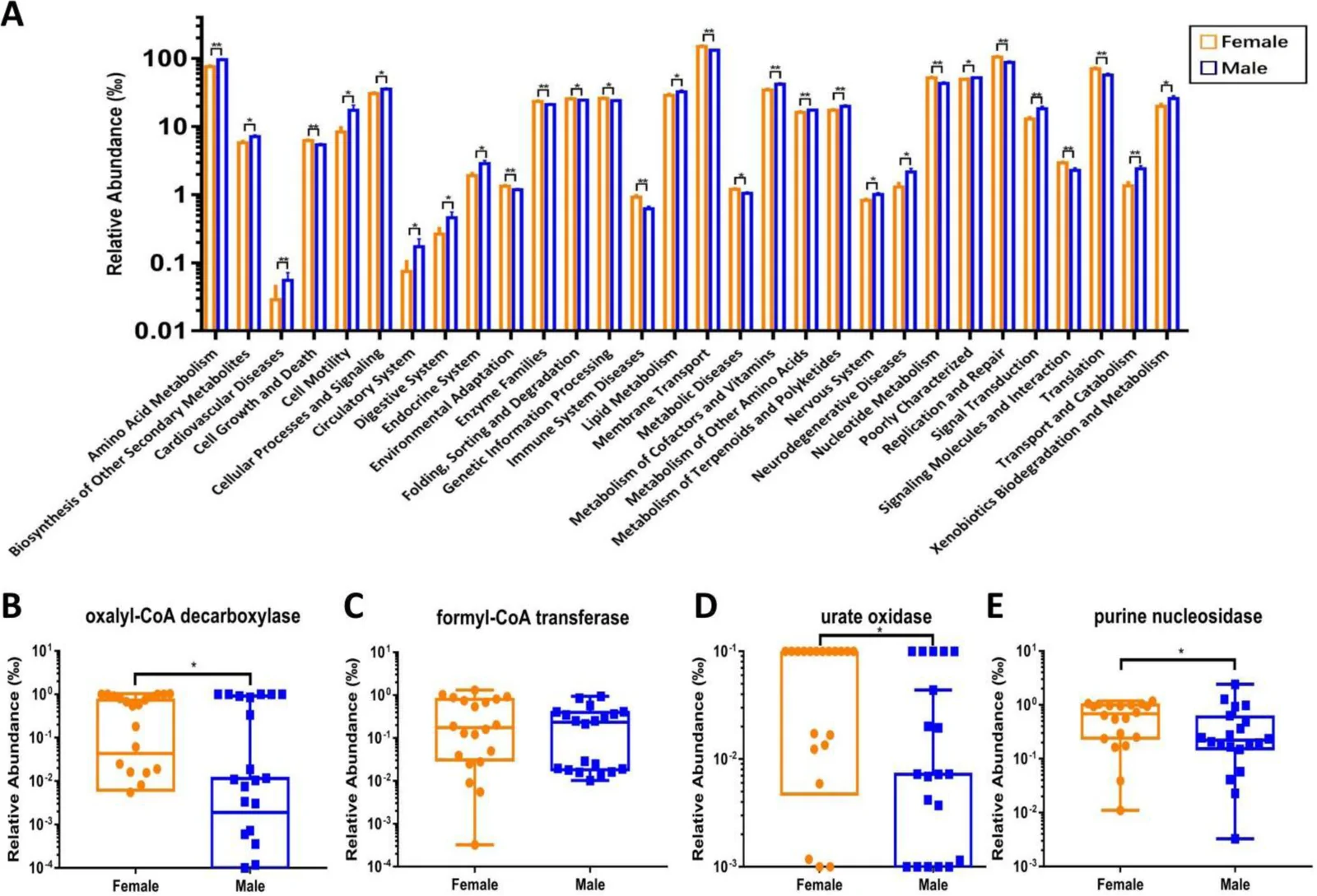
Sex-dependent differences in metabolic functions of the healthy urinary microbiome. A: Comparison of predicted functional pathways at the KEGG second hierarchical level. B–E. Relative abundance (RA) of stone-degrading enzymes in the healthy urinary microbiome: (B) oxalyl-CoA decarboxylase, (C) formyl-CoA transferase, (D) urate oxidase, and (E) purine nucleosidase. *P < 0.05, **P < 0.01.

These results indicate that sexual dimorphism extends beyond taxonomic composition to functional capacity, suggesting that male and female urinary microbiomes may confer different metabolic potentials relevant to urolith formation.

### 3.3. Microbial functional networks reveal a potential protective role in urolith degradation

Given the clinical relevance of urolithiasis, we focused on the predicted functional capacity of the healthy urinary microbiome in mineral degradation. PICRUSt analysis revealed that a series of functional enzymes of urolith degradation existed in the healthy urinary tract, including oxalyl-CoA decarboxylase, formyl-CoA transferase, urate oxidase, and purine nucleosidase. Among them, the highest relative abundance (RA) of purine nucleosidase (0.436‰, 95%CI 0.181‰–0.957‰) was observed in the healthy urinary tract, followed by formyl-CoA transferase (0.175‰, 95%CI 0.021‰–0.578‰), oxalyl-CoA decarboxylase (0.011‰, 95%CI 0.000‰–0.492‰), and urate oxidase (0.000‰, 95%CI 0.000‰–0.007‰). Comparisons between the RAs of different enzymes showed a higher RA of purine nucleosidase than the others (*P* < 0.001) (Figure 4A).

Correlation analysis between 25 key genera and stone-degradation enzymes revealed structured associations (Figure 4B). Among those key microorganisms, only *Lactobacillus* was positively correlated to the generation of oxalyl-CoA decarboxylase and formyl-CoA transferase, indicating the important role of *Lactobacillus* in oxalate degradation. On the other hand, eight key genera were found related to the enzyme generation of uric acid degradation, namely *Enterococcus*, *Lactobacillus*, *Gardnerella*, *Acinetobacter*, *Ochrobactrum*, *Phyllobacterium*, an unclassified genus of *Lactobacillaceae*, and an unclassified genus of *Caulobacteraceae*. As to purine nucleosidase, only *Enterococcus*, *Lactobacillus*, and *Gardnerella* were found to be related to the generation of purine nucleosidase; the other microorganisms were responsible for urate oxidase synthesis, indicating that *Enterococcus*, *Lactobacillus*, and *Gardnerella* were the most important microbes in the degradation of uric acid.

**Figure 4.**
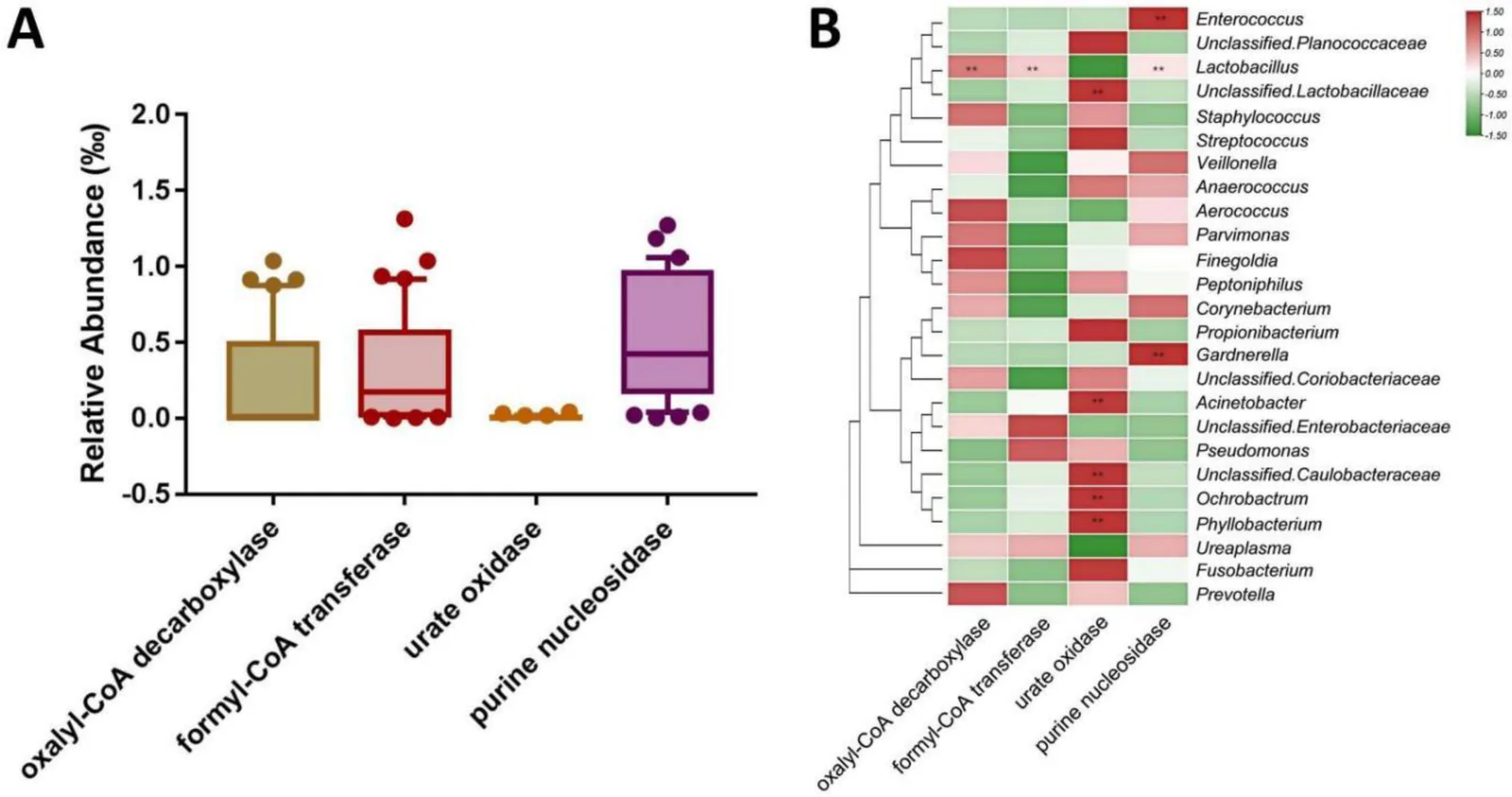
Predicted stone-degrading metabolic potential of the healthy urinary microbiome. A: Relative abundance of stone-degrading enzymes predicted by PICRUSt analysis in the urinary microbiome. B: Correlations between 25 key genera and stone-degrading enzyme levels. The heatmap illustrates correlations between genera and enzyme levels, with red as positive and green as negative correlations. Significant associations are marked with asterisks (*P < 0.05; **P < 0.01).

The integration of co-occurrence network topology with functional predictions suggests that stone-degrading capacity may be an emergent property of microbial consortia rather than individual taxa. This ecological perspective provides a conceptual advance: The urinary microbiome may act as a metabolically cooperative system contributing to mineral homeostasis.

### 3.4. Disease-associated microbial remodeling reveals sex-specific loss of litholytic microbial functions

To determine how urolithiasis alters urinary microbial ecology, we compared microbiomes from healthy individuals and patients. LEfSe analysis identified disease-enriched genera in male and female patients separately (Figure 5A,B), indicating sex-specific microbial shifts in urolithiasis.

**Figure 5.**
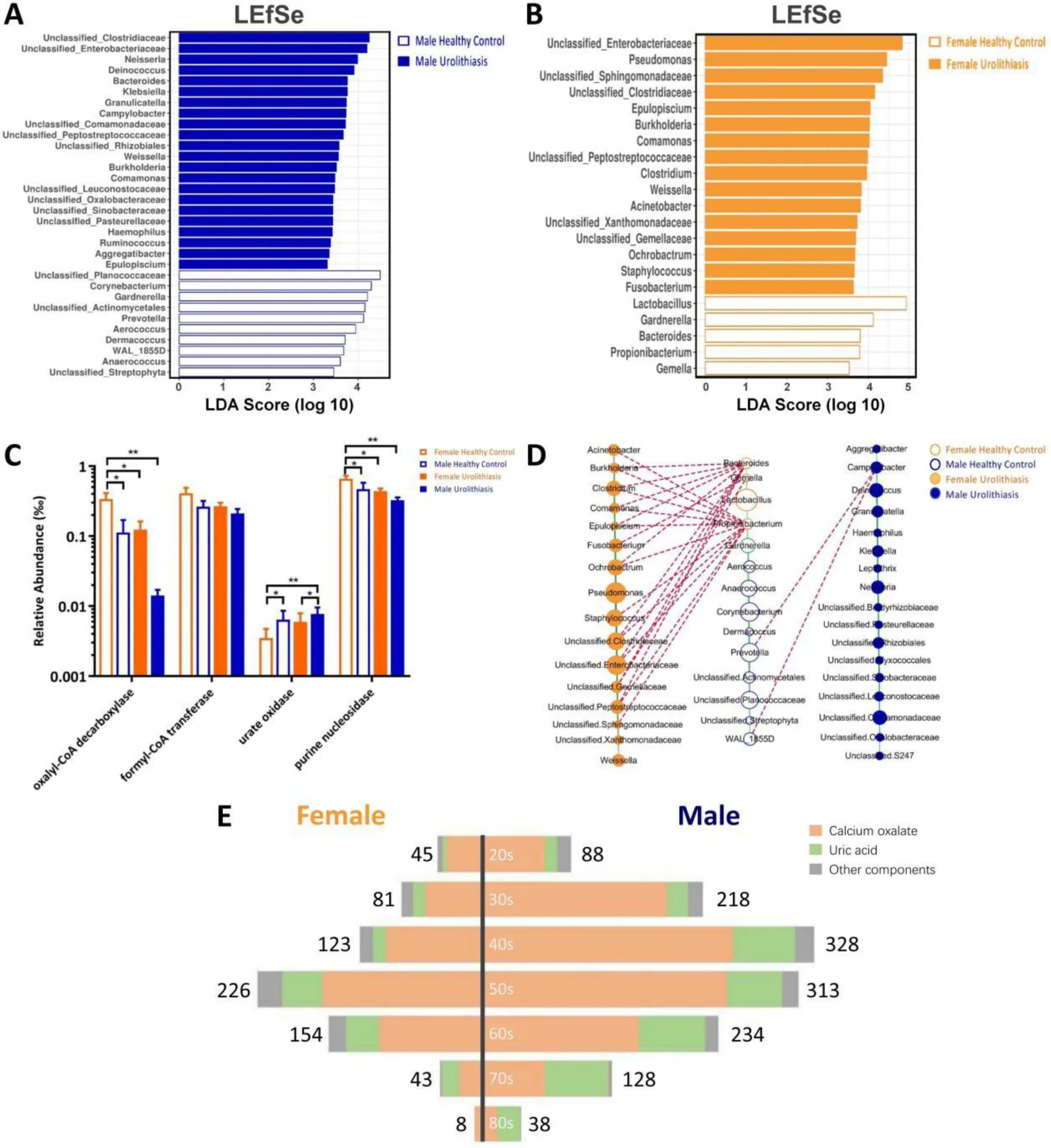
Sex-specific microbial alterations and clinical relevance in urolithiasis. (A and B): LEfSe analysis identifying disease-specific genera in the male (A) and female (B) urinary microbiomes. C: Comparison of the relative abundance of stone-degrading enzymes between healthy controls and urolithiasis patients by sex. D: Co-occurrence networks of dominant urinary genera in healthy individuals and urolithiasis patients. Nodes represent genera (node size indicates relative abundance), and edges represent Pearson's correlations (green, positive; red, negative). E: Retrospective analysis of stone composition across age groups. Calcium oxalate predominated in females at all ages, whereas in males, the dominant composition shifted from calcium oxalate to uric acid in the 70s age group. Age groups: 20s (18–29 y), 30s (30–39 y), 40s (40–49 y), 50s (50–59 y), 60s (60–69 y), 70s (70–79 y), 80s (≥80 y).

Functional comparison revealed a significant reduction in stone-degradation enzyme abundance in urolithiasis samples relative to healthy controls (Figure 5C). Notably, this decline was particularly evident in females, suggesting a pronounced loss of mineral-degrading functional potential in female urolithiasis. Network analysis further demonstrated disruption of microbial ecological structure in disease (Figure 5D). Healthy microbiomes exhibited well-connected networks with identifiable core taxa (yellow circle for female, blue circle for male), whereas urolithiasis-associated networks displayed reduced connectivity and altered interaction patterns. These findings indicate ecological destabilization accompanying disease.

Additionally, retrospective analysis of urolith composition in 2027 patients supports the clinical relevance of these microbial functional shifts: The occurrence of calcium oxalate stone was similar in male and female urolithiasis patients (71.27% vs 70.59%, *P* = 0.750), while the occurrence of uric acid stone was significantly higher in male urolithiasis patients than female urolithiasis patients (22.42% vs 17.79%, *P* = 0.016), with lower occurrence of other-component stones in male than female patients (6.31% vs 11.62%, *P* < 0.001) (Figure 5E).

Together, these results suggest that urolithiasis is characterized by sex-dependent dysbiosis, marked by reduced abundance of mineral-degrading taxa and disruption of microbial ecological networks. This functional loss may contribute to increased stone formation risk.

## 4. Discussion

The urinary tract used to be considered free of pathogens in culture-dependent studies, and those isolated microbes in urine were usually related to the inflammatory pathological state of the urinary tract [18],[19]. In this study, we applied the 16S rRNA amplicon sequencing to investigate the urinary microbiome ecosystem, in hopes of understanding the composition and potential functions of the microbiome in the healthy urinary tract. Similar to the limited research [18],[20],[21], our research revealed that dominant urinary genera were *Lactobacillus*, *Streptococcus*, *Corynebacterium*, *Pseudomonas*, *Gardnerella*, and *Enterococcus*. Moreover, these microorganisms interacted with each other and formed a dynamic ecosystem in the urinary tract. Notably, most of the urinary microbes in our research are oligotrophic and unculturable bacteria, explaining the low isolation rate of microorganisms in healthy urine. Although we have developed a better awareness of the existence of the urinary microbiome, little is known about how these bacteria affect human health.

Unlike intestinal research, culture-independent studies of the urinary microbiome are developing at a slow rate [22]. By now, there are still no unified protocols for DNA extraction and library preparation for urinary microbiome studies. As the healthy urine is low in biomass and high in PCR inhibitors [23],[24], bacterial DNA could be detected only in about 63.9%–92.6% participants, and bias may affect study reliability [25]–[27]. In order to reduce these influencing factors, we improved the biomass of the urine by collecting the first-catch urine in three subsequent days and deactivated the PCR inhibitors by using a DNA extraction kit with inhibitor removal techniques. Our results confirmed that such modifications successfully increased the biomass of the urinary microorganisms and increased the success rate of amplicon library preparation, all of which are important in urinary microbiome studies. In our study, we successfully performed all amplicon sequencing in 40 healthy volunteers and 61 urolithiasis patients, indicating the stability and reliability of the study.

Recent studies have proved that the human microbiome is crucial for maintaining our health by its active metabolites [28],[29]. For instance, *Bifidobacterium*-derived inosine has been shown as a novel immunostimulatory targeting cytotoxic T lymphocyte–associated protein 4 in the gastrointestinal system [30], and *Staphylococcus*-derived antimicrobial peptides can stimulate epithelial cell immunity and prevent pathogen overgrowth in the skin system [31]. However, the molecular mechanisms of the urinary microbiome on the urothelial health have remained unexplored.

Urolithiasis is one of the most common diseases in the urinary tract, affecting 10.6% of the worldwide population [2]. Stones are believed to form in the urinary tract when minerals in urine are at a high concentration [32]. Most calculogenic substances, such as calcium oxalate and uric acid, are diet-derived, and when an overdose of these stone-forming substances is obtained from daily meals, they cannot be degraded by human cells and will accumulate in the urine and gradually form stones [33],[34]. A series of studies have pointed out that, unlike human cells, bacterial cells can synthesize a series of active enzymes, such as oxalyl-CoA decarboxylase, formyl-CoA transferase, urate oxidase, and purine nucleosidase, which can decompose the calculogenic substances in the intestinal tract [6],[35],[36]. In our research, we have also discovered a series of mineral-degradative microorganisms in the urinary tract, indicating that these urinary microbes, such as *Lactobacillus*, *Enterococcus*, and *Gardnerella*, may serve as protectors for urolith formation.

Another finding of our research is the different composition and function of the urinary microbiome in different sexes. Although sex disparities in the urinary tract are well documented [8], research about the differences in male and female urinary microbiomes remains scarce. Our study found that *Pseudomonas* and *Streptococcus* were the dominant genera in male samples, while *Lactobacillus* and *Streptococcus* dominated in female samples. These structural disparities lead to functional differences in microorganisms: The RAs of oxalyl-CoA decarboxylase, urate oxidase, and purine nucleosidase were significantly lower in the male urinary microbiome, increasing the risks of stone formation in men. This hypothesis was corroborated by our retrospective analysis of 2027 urolithiasis patients, revealing a male-to-female incidence ratio of 1.98. Even in the elderly, without the protection of sex hormones [9],[37], the incidence remains higher in males, indicating that bacterial differences independently influence urolith formation. Although stone composition was not determined in the sequenced individuals, our 2027-patient cohort provides robust epidemiological support. Specifically, the significantly higher clinical prevalence of uric acid stones in males perfectly parallels their lower RA of uric acid–degrading enzymes compared to females, suggesting a direct link between microbial enzymatic activity and specific stone formation. This enhanced microbial litholytic potential in females is likely driven by anatomical factors. The shorter female urethra and its close proximity to the vagina facilitate continuous microbial exchange, creating a vaginal–urinary axis. This mechanism naturally seeds the female lower urinary tract with dominant vaginal taxa like *Lactobacillus* and *Gardnerella*, establishing a more robust defense system against lithogenic substrates than in males.

To further understand the sex-specific roles of the urinary microbiome in urolithiasis, we investigated the microbial features in male and female patients. Our results revealed a decrease in *Corynebacterium* and *Prevotella* and an increase in *Neisseria* and *Enterococcus* in male urolithiasis, as well as a decrease in *Lactobacillus* and *Gardnerella* and an increase in *Enterobacter* and *Pseudomonas* in female urolithiasis. These compositional shifts in females led to a predicted loss of oxalyl-CoA decarboxylase and purine nucleosidase, potentially increasing uric acid stone risks, whereas the male functional profile remained unchanged. Moreover, co-occurrence analysis revealed that in female urolithiasis, pathogens exhibit agonistic effects on protective microbes; this correlation was less evident in males. This highlights the importance of maintaining microbial homeostasis in females, where pathogen overgrowth may suppress beneficial taxa and weaken stone protection. Importantly, the enzymatic capacities reported herein are inferred from 16S rRNA sequencing via PICRUSt, representing potential rather than confirmed activity. While our study focuses on the loss of protective litholytic potential, dysbiosis likely also introduces stone-forming activities (e.g., urease from *Proteus* promoting struvite, or citrate lyase from *E. coli* depleting citrate). Rather than negating our findings, this suggests that microbial dysbiosis in urolithiasis is a “dual-hit” process: A significant loss of protective bacteria coupled with an overgrowth of stone-promoting taxa. Future studies employing shotgun metagenomics or metatranscriptomics are essential to definitively validate these metabolic functions and fully characterize this complex microbial interplay.

**Figure 6.**
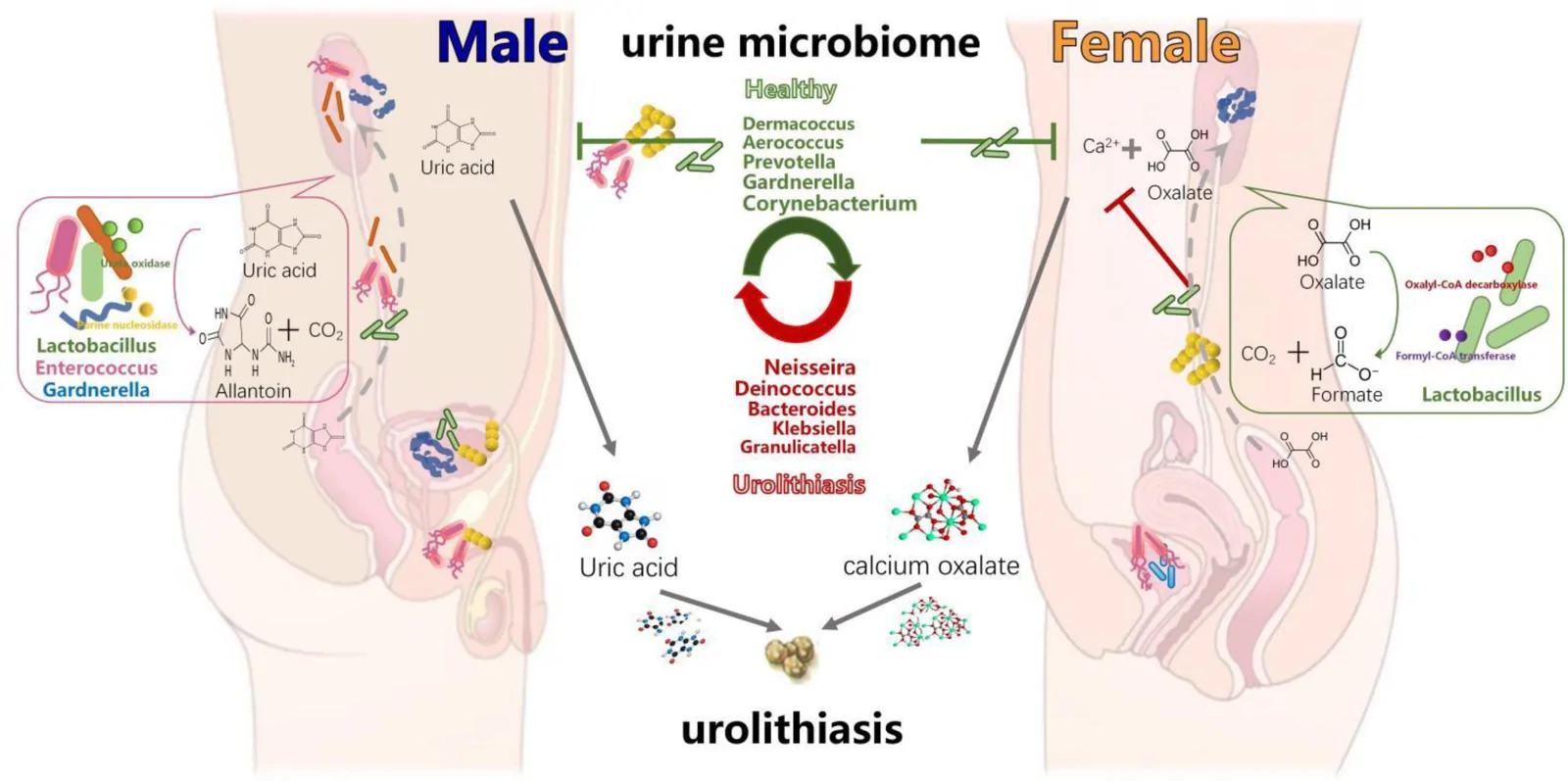
Proposed schematic of microbial dynamics in the urinary tract during urolithiasis development. A diverse microbial community is present in the healthy urinary tract, and its composition shifts with the onset and progression of urolithiasis. Urinary *Lactobacillus* may contribute to protection against calcium oxalate stone formation through the production of oxalyl-CoA decarboxylase and formyl-CoA transferase. In addition, *Lactobacillus*, *Enterococcus*, and *Gardnerella* may participate in uric acid degradation via urate oxidase and purine nucleosidase production.

## 5. Conclusions

Our study offers a set of reliable protocols for urinary microbiome analysis by collectively collecting three days of first-catch urine and extracting microbial DNA with an inhibitor removal technique. Using these protocols, we obtained detailed composition and functional characteristics of the healthy urinary microbiome and validated the different risks of urolith formation brought by the sex-specific microbiome. Our findings also indicate that a balance in the urinary microbiome is crucial in preventing diseases such as urolithiasis. Overall, our results provide foundations for novel microbiome-based diagnostics and therapeutics for urinary diseases.

## Use of AI tools declaration

The authors declare they have not used Artificial Intelligence (AI) tools in the creation of this article.

## Conflict of interest

All the authors declared no competing interests.

## Author contributions

YL, CM, RW and QW conceptualized and designed the study. CM, YX and SJ recruited the clinical cohort and collected samples. YX, LC and JL performed the retrospective study. YL, ZH and TZ contributed to the amplicon sequencing. YL, SH and XX analyzed the data, YL, YX and JZ wrote the manuscript; CM, RW and QW performed critical revisions of the manuscript.
